# Supplementary material for: Nanoconfinement enabled non-covalently decorated MXene membranes for ion-sieving
Source: Nat Commun. 2023 Jul 10;14:4075. doi: 10.1038/s41467-023-39533-y (PMC10333198; doi:10.1038/s41467-023-39533-y)
Supplement: Supplementary file 1 — Supplementary Information [file 41467_2023_39533_MOESM1_ESM.docx]

**Nanoconfinement Enabled Non-covalently Decorated MXene Membranes for Ion-sieving**

Yuan Kang^1^, Ting Hu^1^, Yuqi Wang^2^, Kaiqiang He^1^, Zhuyuan Wang^1^, Yvonne Hora^1^, Wang Zhao^1^, Rongming Xu^3^, Yu Chen^4^, Zongli Xie^5^, Huanting Wang^1^, Qinfen Gu^6^*, Xiwang Zhang^1,7^*

1. Department of Chemical and Biological Engineering, Monash University, 3800, Australia

2. School of Materials Science and Engineering, Zhejiang University, 310058, China

3. School of the Environment, Nanjing University, Nanjing, 210023, China

4. Monash Centre for Electron Microscopy, Monash University, 3800, Australia

5. CSIRO Manufacturing, Private Bag 10, Clayton South, 3169, Australia

6. Australian Synchrotron (ANSTO), Clayton, 3168, Australia

7. UQ Dow Centre for Sustainable Engineering Innovation, School of Chemical Engineering, The University of Queensland, St. Lucia, 4072, Australia

* Email: xiwang.zhang@uq.edu.au; qinfeng@ansto.gov.au

**Supplementary Fig. 1. Morphology characterizations of Ti_3_C_2_T_x_ nanosheets, including (a) Transmission electron microscopy (TEM) image (inset, diffraction pattern) and (b) Atomic force microscopy (AFM) height profile (inset, nanosheets from a different sample).**

Ti_3_C_2_T*_x_* nanosheet deposited on carbon film showed a flat morphology with very high degree of transparency. Its diffraction pattern presented a clear typical monocrystalline hexagon lattice, indicating the monolayered nature of the nanosheet (Fig S1 a). This was further supported by the AFM height profile of the nanosheets between 1.2 to 1.5 nm (Supplementary Fig. 1b and inset). The measurement was slightly higher than theoretically predicted Ti_3_C_2_T*_x_* thickness (0.88 to 0.98 nm) due to adsorbed impurities such as CO_2_ and H_2_O^1,2^. Meanwhile, the smooth nanosheet edges, and the relatively low nanosheet-mica contrast between indicated the negligible presence of oxidized parts that would have otherwise caused higher contrast due to their larger size. Together with the clear TEM diffraction pattern, such AFM features can imply the low oxidation degree of the prepared MXene nanosheets.


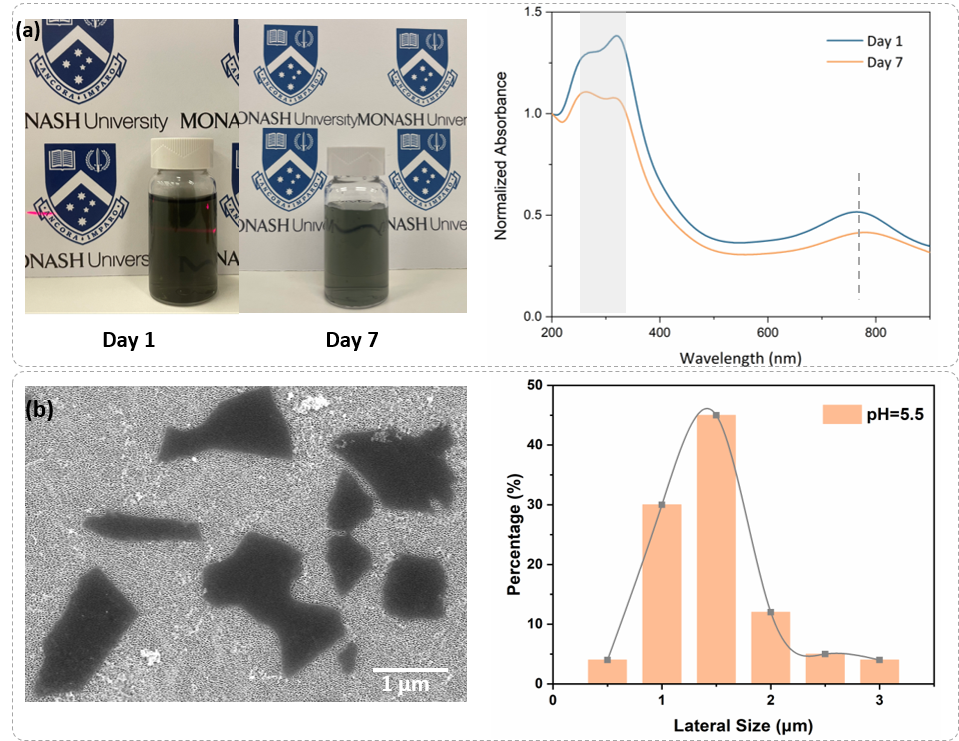


**Supplementary Fig. 2. Stability and size distribution of dispersed Ti_3_C_2_T_x_ nanosheets. (a) Ti_3_C_2_T*_x_* dispersion at room temperature and its UV-vis spectra over a week time. The shaded regions and dashed lines highlight UV-vis peak changes caused by MXene oxidation. (b) Dispersed Ti_3_C_2_T*_x_* nanosheets deposited on AAO support and their lateral size distribution (by counts).**

Freshly prepared (Day 1) Ti_3_C_2_T_x_ dispersion exhibited typical “Tyndall effect” (Supplementary Fig. 2a), and its corresponding UV-vis spectra showed an absorption peak at 770 nm, the intensity of which is commonly used to calculate dispersion concentration and to monitor the changing oxidation status of the dispersed Ti_3_C_2_T*_x_*^3^. As can be seen, the dispersion started turning cloudy white from Day 7, suggesting the transition of part of Ti_3_C_2_T_x_ into TiO_2_. Meanwhile, peak intensity at 770 nm decreased, further proving the ongoing material oxidation^3^. All the experiments in this work were thereby finished with fresh MXene dispersion. When deposited on porous anodized aluminum oxide (AAO), the nanosheets allowed the measurement of lateral size (Supplementary Fig. 2b). Counting over 200 pieces of nanosheets gave a relatively narrow size distribution centering around 1.5 μm.


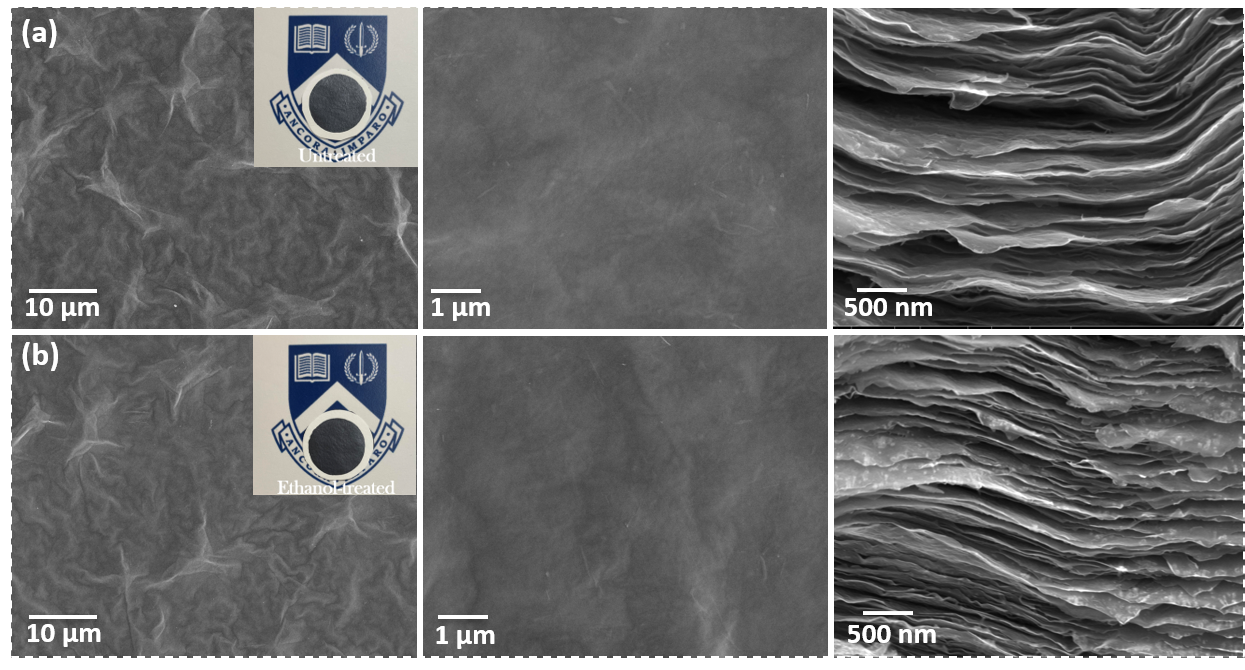


**Supplementary Fig. 3 SEM morphological comparison of Untreated-M (a) and EtOH-M (b) in low resolution front surface mode (Left, scale bar 10 μm, inset: membrane digital photos), high resolution front surface mode (Middle, scale bar 1 μm) and cross-section mode (Right, scale bar 500 nm).**

MXene membranes were assembled by filtrating Ti_3_C_2_T*_x_* nanosheets on Nylon substrate for digital and SEM photo shooting. After drying, both Untreated-M and EtOH-M showed continuous and undulated surface in low resolution SEM mode, a typical feature to 2D material-based materials. High resolution scanning on them revealed their defect-free surface and layered cross-sectional structure. Meanwhile, these highly similar morphological features prove that post-assembly solvent treatment hardly alters the structure of Ti_3_C_2_T*_x_* membranes.


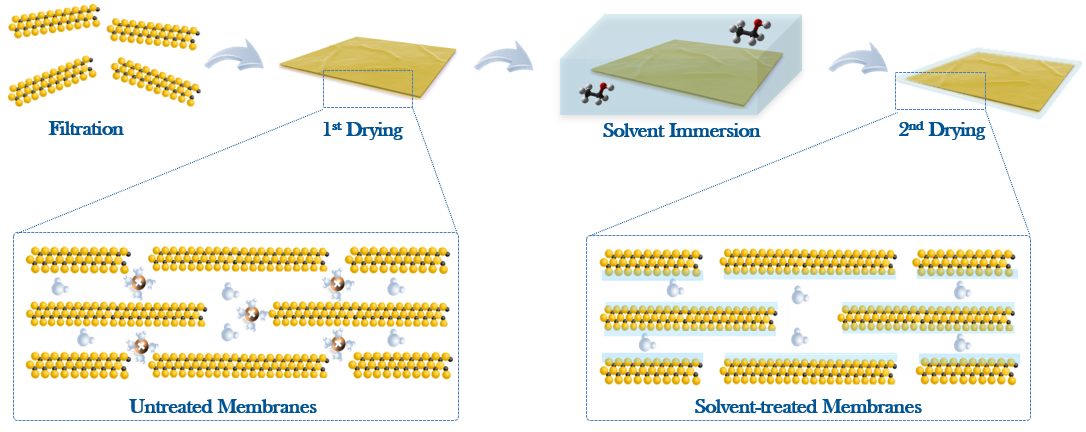


**Supplementary Fig. 4. Scheme for the preparation of solvent-treated membranes.**

The preparation of solvent-treated membranes was achieved by a simple solvent immersion operation. An untreated membrane was assembled via vacuum-assisted filtration method and dried for 24 hours. The membrane was then immersed in solvents (either ethanol, acetone or cyclohexane) for at least 2 hours to ensure impregnation to the most possible degree. The treated membrane was then transferred into a fan-forced oven (50℃, 2 hours) where it was dried for a 2^nd^ time to fully evaporate these highly volatile solvents. The as-prepared solvent-treated membranes were then used for further performance testing and characterizations.


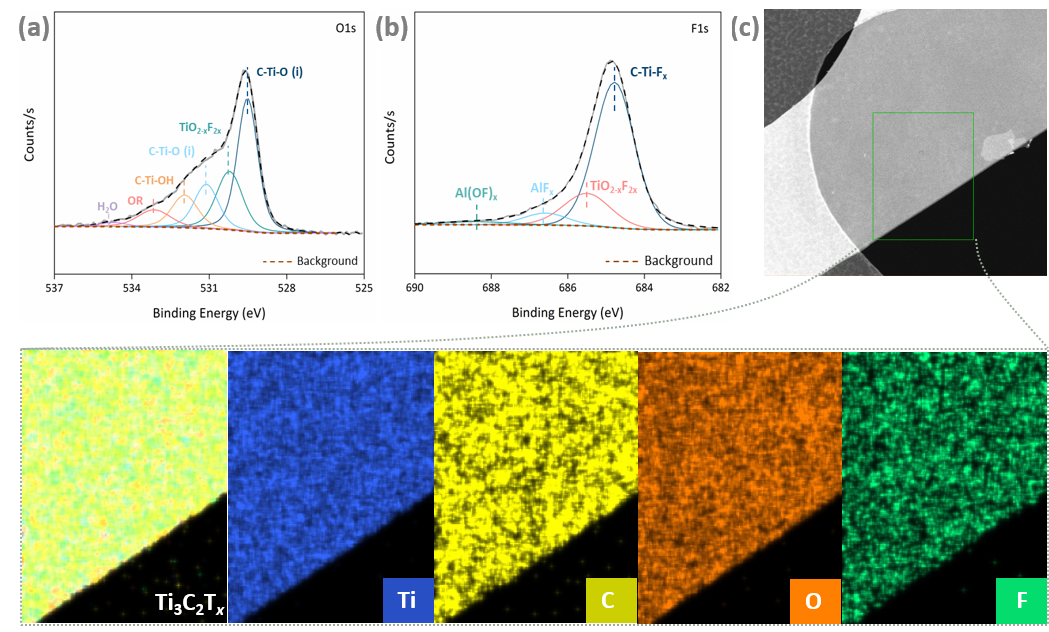


**Supplementary Fig. 5. X-ray photoelectron spectroscopy (XPS) spectra of MXene membranes including (a) O1s and (b) F1s. TEM image of MXene (c) and associated energy Dispersive X-ray (EDX) microanalysis.**

The O1*s* spectra can be decomposed into several peaks at 529.5 eV for C-Ti-O (i), 530.2 eV for TiO_2-x_F_x_, 531.1 eV for C-Ti-O (ii), 532.0 eV for C-Ti-OH, 533.2 eV for -OR and 534.7 eV for H_2_O. The F1*s* spectra can be decomposed into peaks at 684.8 eV for C-Ti-F, 685.5 eV for TiO_2-x_F_x_, 686.6 eV for AlF_x_, 688.4 eV for Al (OF)_x_. After omitting the unnecessary species (such as H_2_O, Al(OH)_x_ and AlF_x_) and dropping out the oxidized part, we could obtain the simplified MXene molecular formula of Ti_3_C_2_O_0.66_(OH)_0.18_F_0.51_. Energy Dispersive X-ray (EDX) microanalysis in selected area (c) show that the main composition elements of MXene including Ti, C, O and F are evenly distributed across its whole surface. This indicates that functional groups attached to the MXene are also well spread not only in the basal plane, but also along the edge of MXene nanosheets.

**Supplementary Fig. 6. XRD spectra of Untreated-M and EtOH-M in both dry and hydrated conditions.**

The XRD spectrum of Untreated-M of EtOH-M in dry status showed an outstanding (002) peak at around 6.5°, pointing to *d*-spacing of 13.7 Å. When immersed in water, they exhibited again highly similar (002) peak at around 5.4°, indicating moderately enlarged *d*-spacing of 16.5 Å due to hydration. Meanwhile, it is worth noting that, in both dry and hydrated conditions, the rest of XRD spectra (7° to 70°) for EtOH-M is also almost identical to that for Untreated-M. This implies that moderate heating of EtOH-M at 50°C will not cause any substantial structural or chemical changes to the membranes.

**Supplemental Table 1. Modification efficacy comparison of EtOH-M with recently reported membranes based on their ion sieving performance.**

**Membrane Thickness (μm) Ion feed Ion PR* (mmol·hr^-1^·m^-2^) Enhancement Ref.**

**Materials channel size (Å) concentration (M) before/after treatment factor**

Ti_3_C_2_T*_x_* 0.5 (5.6) 0.2 (Na^+^) 222 / ~12 19 Ref.4

(Ti-O-T) 0.5 (5.5) 222 / ~8 27

0.5 (5.4) 222 / 6.7 33

Ti_3_C_2_T*_x_* 0.34 (5.6) 0.2 (Na^+^) ~420/~60 7 Ref.5

(Al^3+^-O) 0.58 (5.6) ~230/~18 13

1.1 (5.6) ~112/~4.5 25

Ti_3_C_2_T*_x_* 5.0 (2.7) 0.5 (Na^+^) ~48/~12 4 Ref.2

(Al_13_-O) 5.0 (4.7) ~48/~35 1.4

Ti_3_C_2_T*_x_* 2.0 (6.5) 0.2 (Li^+^) 283/80 3.5 Ref.6

(Ti-O-Ti & -SO_3_H)

GO 0.2 (10.1) 0.2 (Na^+^) ~180 / ~35 5.1 Ref.7

(-NH_2_&-N) 0.2 (9.8) ~180/ ~3.5 51

0.2 (10.1) 0.2 (K^+^) ~230/ ~55 4.2

0.2 (9.8) ~230/ ~3.8 61

GO >1.0 (5.6) 1.0 (Na^+^) ~5 / ~1.2 4.2 Ref.8

(epoxy >1.0 (5.2) ~5 / ~0.65 7.7

Confinement) >1.0 (4.5) ~5 / ~0.35 14.3

>1.0 (4.2) ~5 / ~0.17 29.4

GO (EDA) ~22 (7.5) 0.1 (Na^+^) ~113 / ~65 1.7 Ref.9

Ethylenediamine

GO (PPD) ~22 (7.2) ~113 / ~13 8.7

p-Phenylenediamine

GO (OPD) ~22 (7.7) ~113 / ~8 14.1

o-Phenylenediamine

Ti_3_C_2_T*_x_* 300 (< 7.7) 0.2 (Na^+^) 368/15.8 23.3 This

(EtOH) 300 (< 7.7) 0.2 (Li^+^) 334/14.3 23.4 work

300 (< 7.7) 0.2 (K^+^) 377.5/25.4 14.9

***PR: Permeation rate**

**
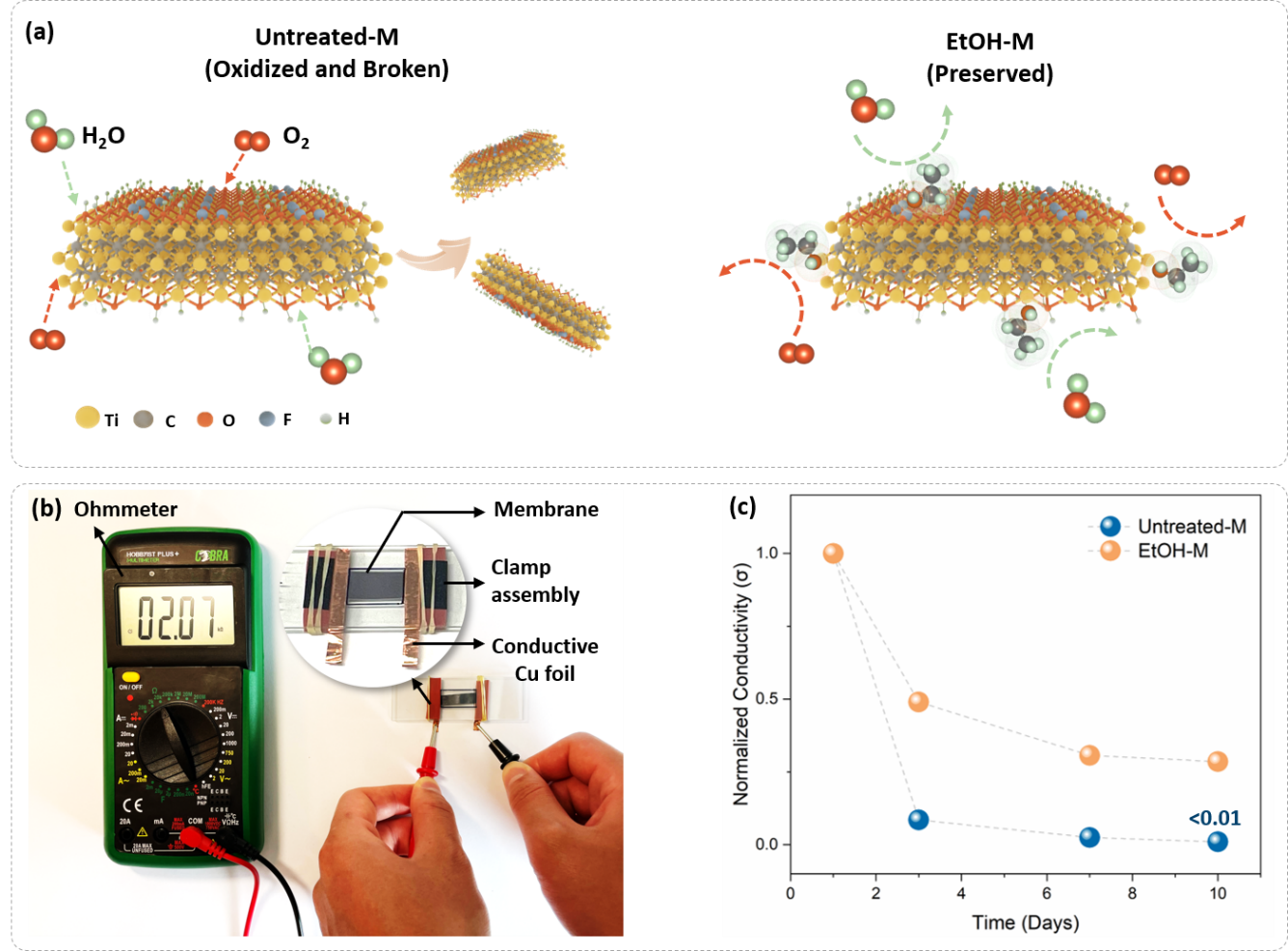
**

**Supplementary Fig. 7. (a) Comparison of the anti-oxidation ability of Untreated-M and EtOH-M in aqueous conditions by (b) conductivity test. The normalized results are shown in (c).**

Unwanted oxidation of MXene materials including Ti_3_C_2_T*_x_* is a universal problem in either dispersion or solid form (e.g., layered membranes). This happens when reactive H_2_O and O_2_ attack the Ti-C bonds of Ti_3_C_2_T*_x_* nanosheets, resulting in their de-functionalization and breaking. However, ethanol molecules attached onto the Ti_3_C_2_T*_x_* will prevent the direct contact of the material with reactants, which creates a “shield” to largely slow down the oxidation process (Fig. S7a)^10,11^. To prove this proposed mechanism, we designed a proof-of-concept experiment based on membrane conductivity (*σ*) testing, which is consider a reliable indicator of solid MXene oxidation degree (lower *σ* implies higher oxidation)^11,12^. The testing was carried out using a homemade assembly where the Untreated-M or EtOH-M was firmly clamped and attached to conductive Cu foils for consistent measurement (Fig. S7b). Both assemblies were kept in water, taken out and thoroughly dried for resistance (*R*) measurement at the 1st, 3rd, 7th, and 10th day, and *σ* can be calculated by the equation below:

$\sigma=\frac{L}{R\cdot A}$ (1)

where *L* and *A* respectively represents tested membrane length and cross-sectional area, and further simplified as:

$\sigma=\frac{1}{R}$ (2)

, since *L* and *A* remained unchanged throughout the experiment. On this basis, *σ* measured at each testing point was normalized against its initial *σ_0_* and plotted in Fig. S7c. It can be seen that both membranes experienced conductivity loss over the 10-day period, indicating ongoing MXene oxidation. However, such loss in Untreated-M was significantly faster, with conductivity plummeting to below 10% in 3 days, than in EtOH-M whose conductivity could maintain above 50% of its initial value. Although a fully quantitative link can yet be established between conductivity loss and ion permeation increase, we believe that the above comparison well verifies the enhanced resistance of EtOH-M against oxidation.

**
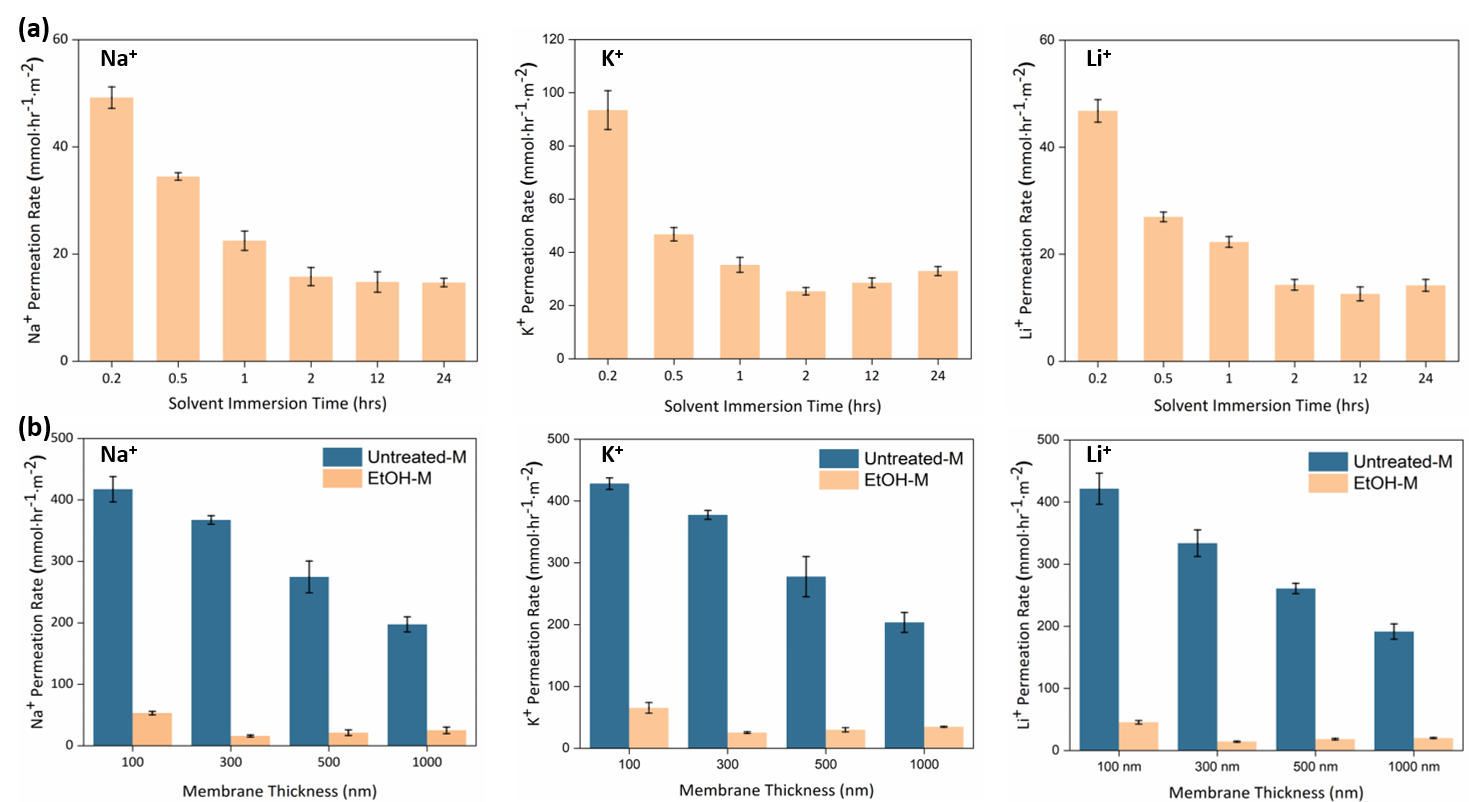
Supplementary Fig. 8. Dependence of EtOH-M ion sieving ability on solvent treatment time (a) and membrane thickness (b). The dependence was evaluated on Na^+^, K^+^ and Li^+^, from left to right. The error bars in this figure represent the standard deviations of three parallel tests.**

The effect of solvent treatment time on membrane Na^+^ sieving capability shows that the intake of ethanol molecules into the membrane reached its limit approximately after 2 hours (Figure S7 a). This is much short than the time (24 hours) taken to complete MXene membrane decoration by hydrated ions, indicating the relatively smaller intake of ethanol than ions. Moreover, it is found that the thickness increase of membrane from 100 nm to 300 nm will boost its ion rejection, while thicker membranes (>300 nm) does not guarantee better ion-blocking ability. This also implies that the impregnation of ethanol into inner layers of MXene membranes is impeded so that the decoration cannot occur throughout the membrane especially in when the membrane becomes thicker. Same tests were also conducted on K^+^ and Li^+^, which showed similar trends.


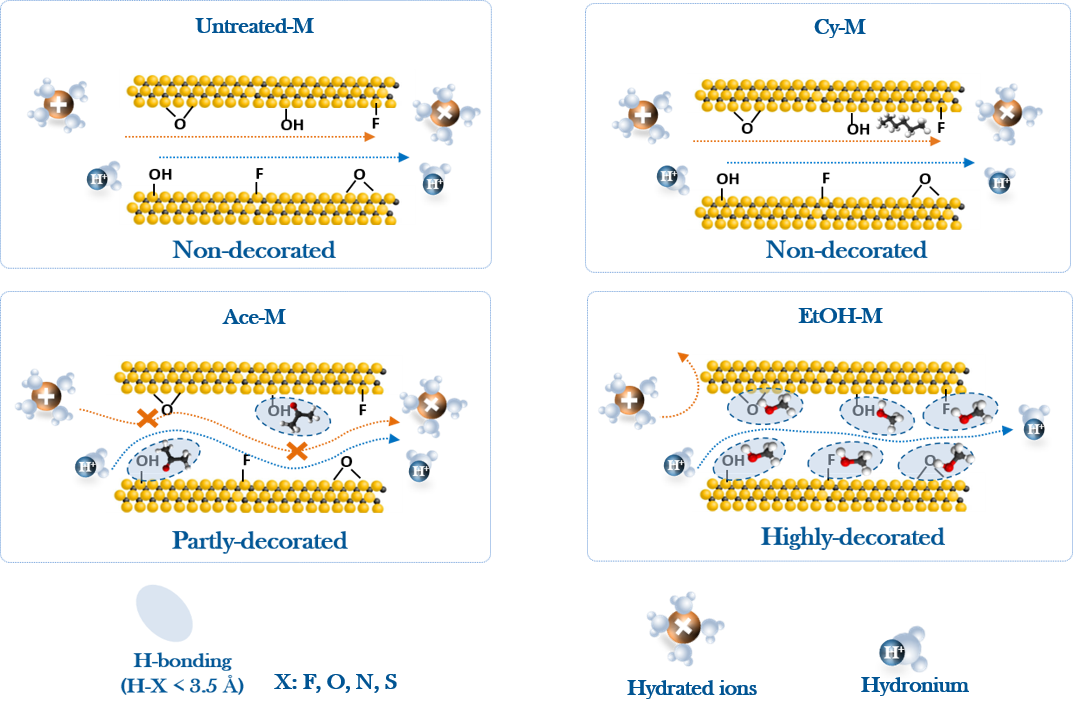


**Supplementary Fig. 9. Schematics of hydrated ions permeating Untreated-M, Cy-M, Ace-M and EtOH-M.**

The effective establishment of H-bond between MXene functionalities and appropriate solvents will lead to MXene channel decoration to varying degree. Because of the protic and polar nature of ethanol molecules, more of them can be attached into the channel than acetone and cyclohexane. Therefore, EtOH-M show much higher ion-sieving and proton-separating ability than Untreated-M, Cy-M and Ace-M.





**Supplementary Fig. 10. Na^+^ sieving and H^+^/Na^+^ selective performance of various membranes including Untreated-M, Hex-M. MeCHO-M and MeOH-M. The error bars in this figure represent the standard deviations of three parallel tests.**

Additional MXene membranes were treated by methanol (MeOH), Acetaldehyde (MeCHO) and n-Hexane (Hex) and tested on their ion rejecting and selective performance. Similar to their counterparts, Hex-M, MeCHO-M and MeOH-M could improve Na^+^ sieving ability by 1.3, 3.9 and 25.3 times compared to Untreated-M, respectively. Accordingly, the H^+^/Na^+^ selectivity was increased from 1.8 to 1.9, 4.1 and 11, respectively.

**Supplementary Table 2. Binding Energy of “One-to-one” pair between solvents (ethanol, acetone and cyclohexane) and functional groups on MXene (-OH, -O and -F).**

| 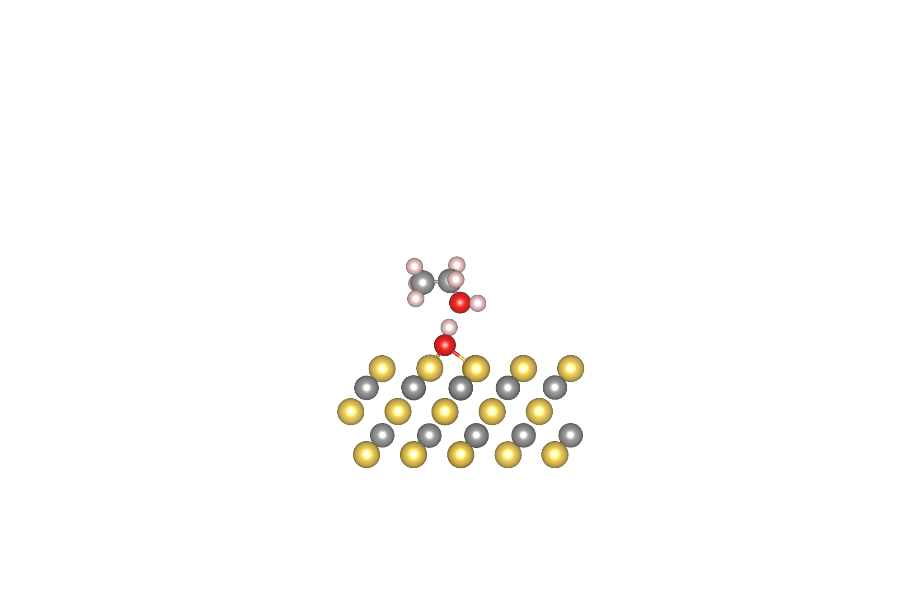  EtOH-OH  E_abs_: -0.479 eV | 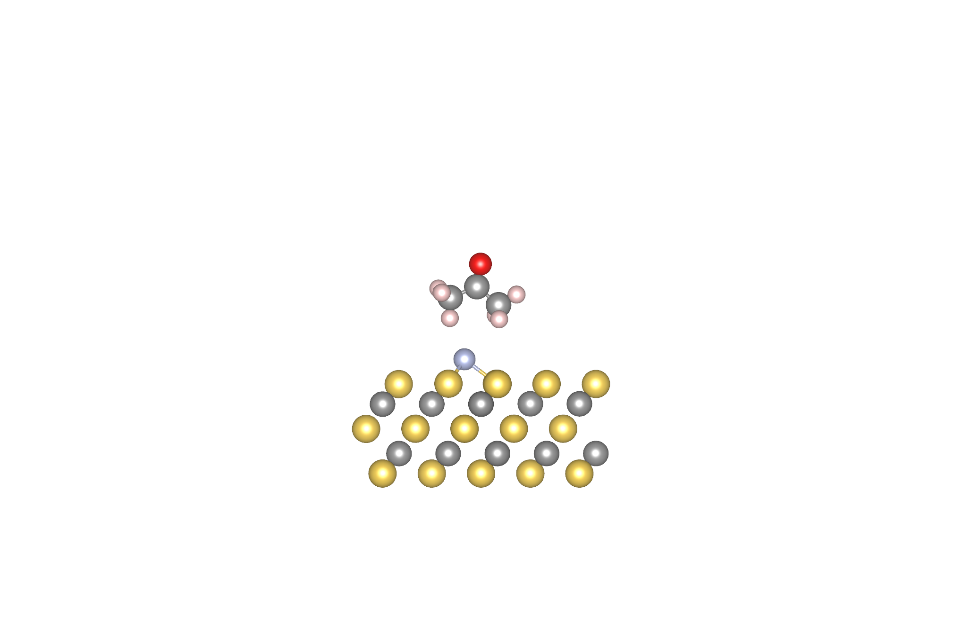  Ace-F  E_abs_: 0.035 eV |
| --- | --- |
| 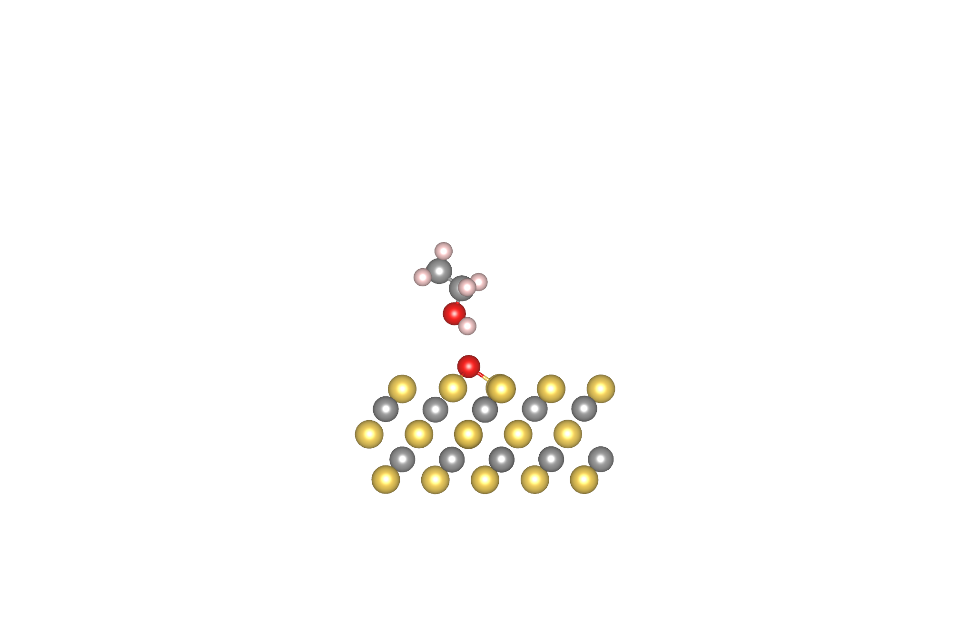  EtOH-O  E_abs_: -0.110 eV | 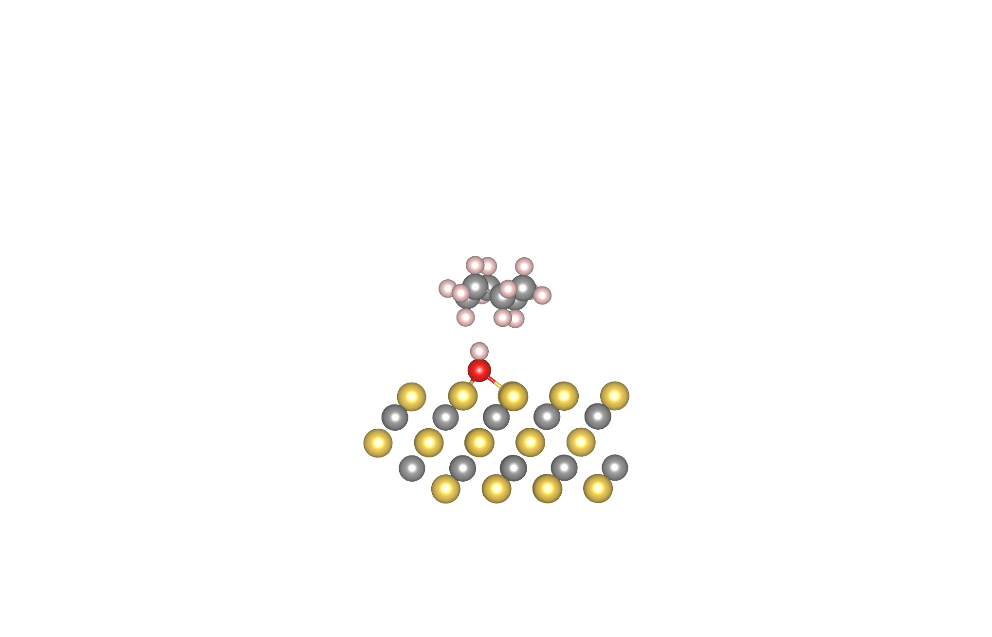  Cy-OH  E_abs_: -0.063 eV |
| 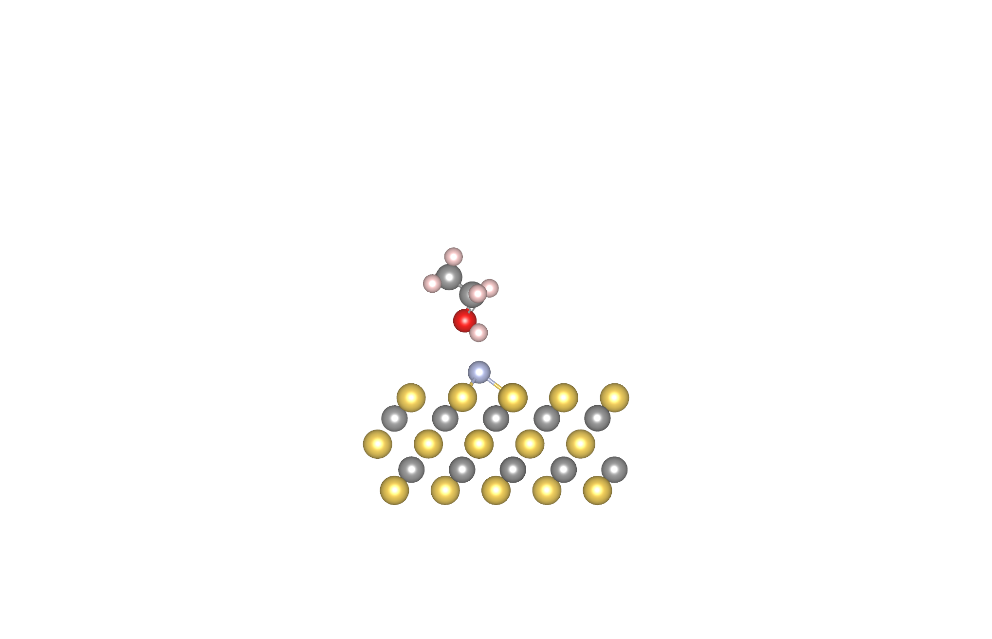  EtOH-F  E_abs_: -0.028 eV | 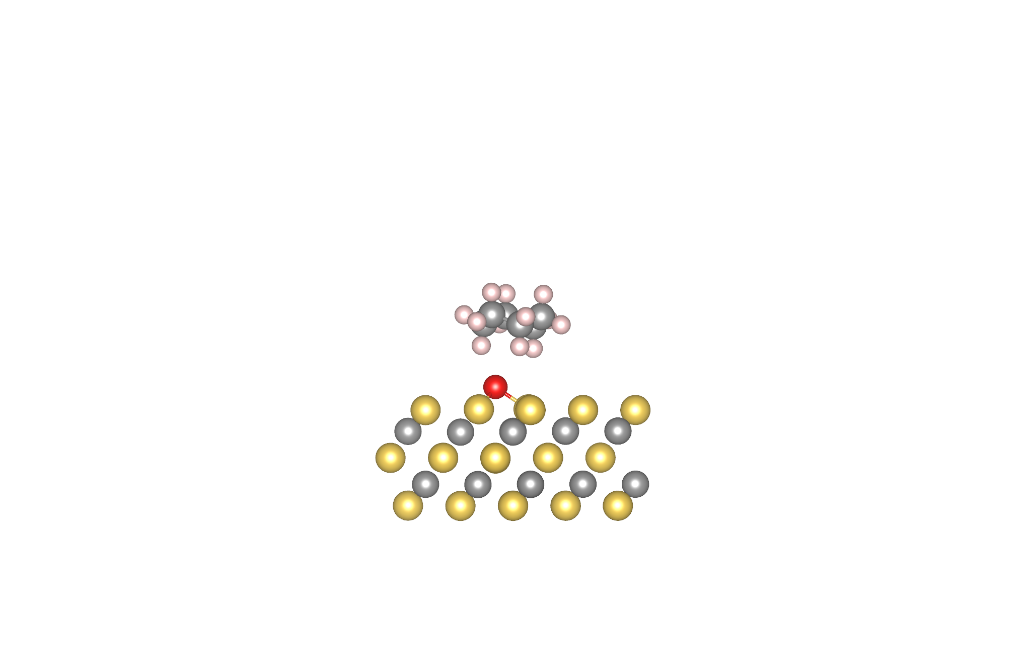  Cy-O  E_abs_: 0.032 eV |
| 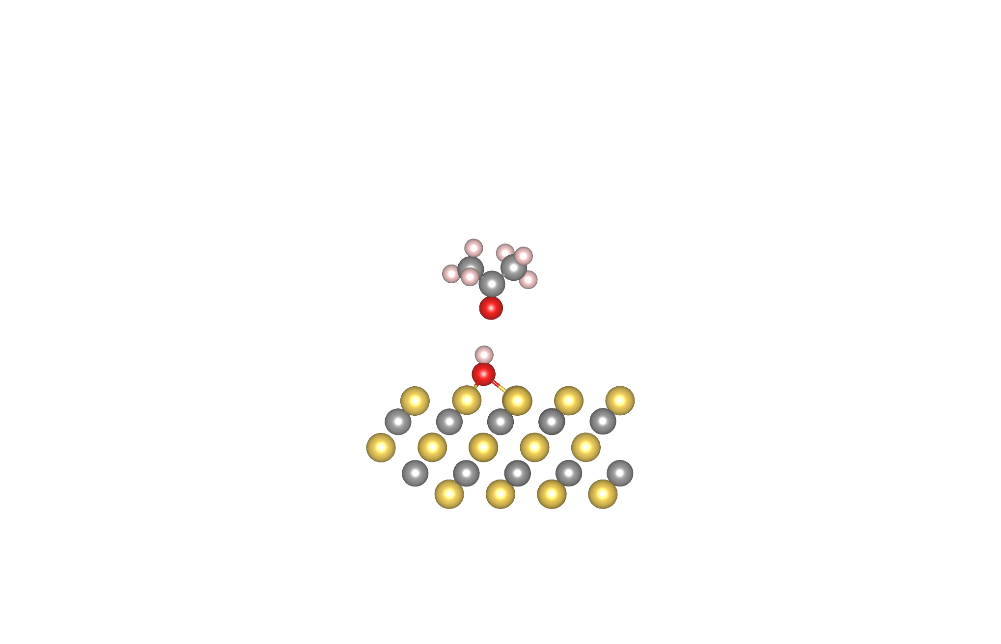  Ace-OH  E_abs_: -0.216 eV | 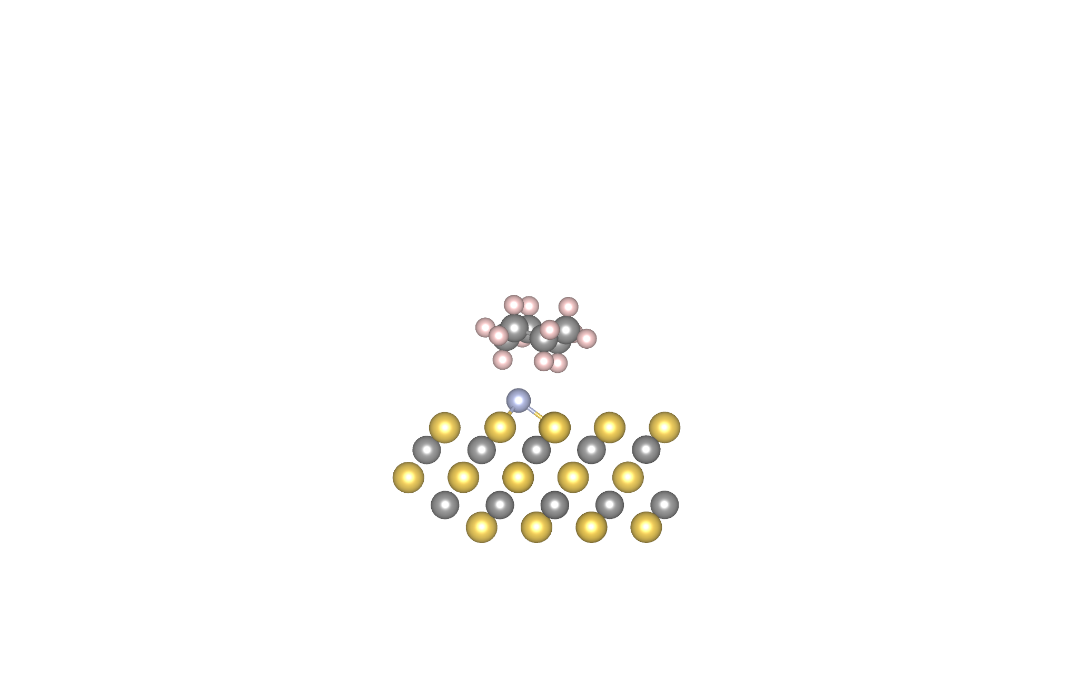  Cy-F  E_abs_: 0.043 eV |
| 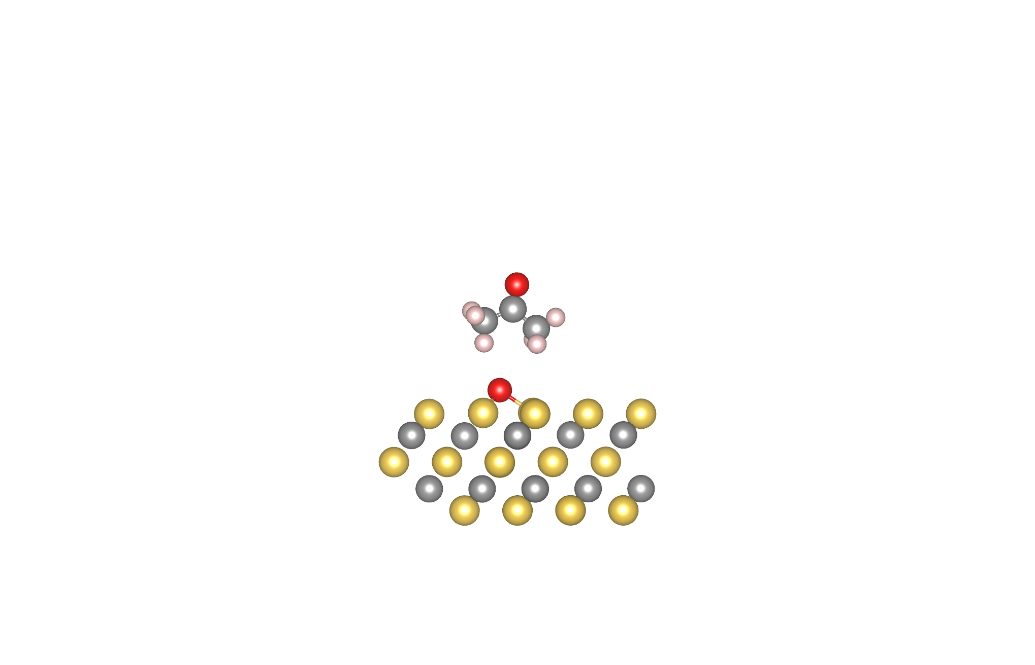  Ace-O  E_abs_: 0.017 eV |  |

**Supplementary Table 3. Geometry and binding energy comparisons of “One-to-one” and “One-between-two” configurations between ethanol and MXene functional groups (-OH, -O and -F).**

| One-to-one mode | One-between-two mode | Enhancement Factor |
| --- | --- | --- |
| 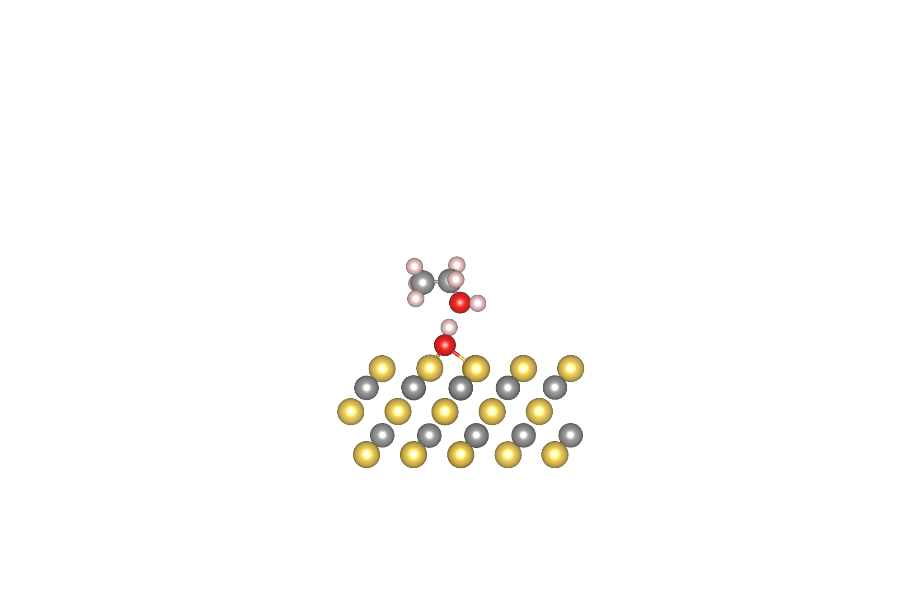  EtOH-OH (-0.479 eV) | 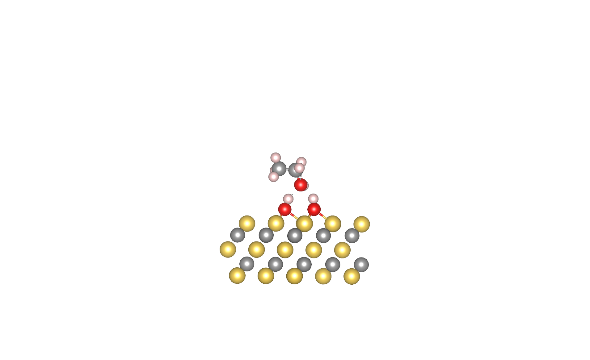  OH-EtOH-OH (-1.290 eV) | 2.7 |
|  | 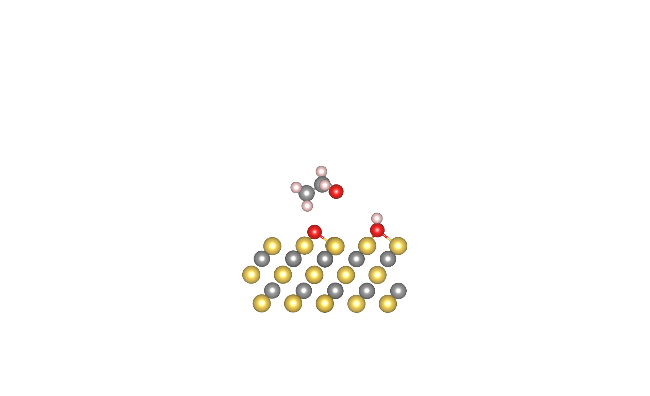  O-EtOH-OH (-0.890 eV) | 1.9 |
|  | 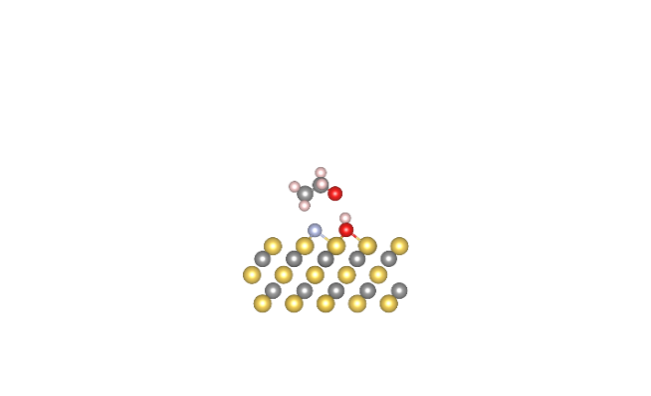  F-EtOH-OH (-0.990 eV) | 2.1 |
| 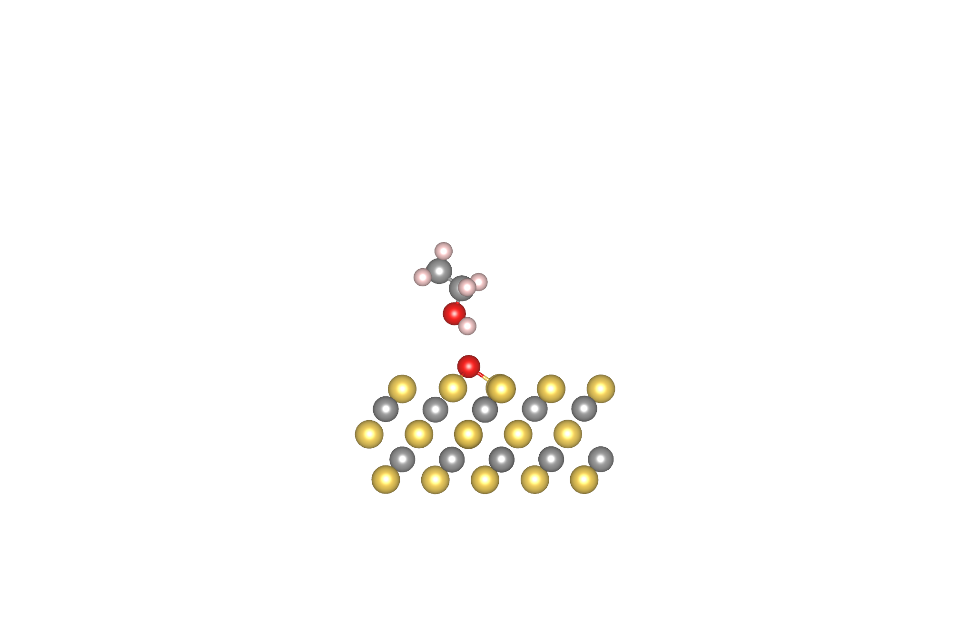  EtOH-O (-0.110 eV) | OH-EtOH-O (-0.890 eV)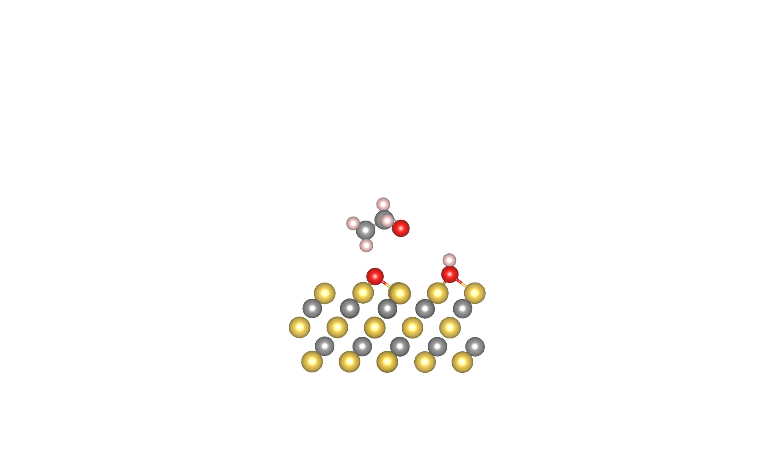 | 8.1 |
|  | 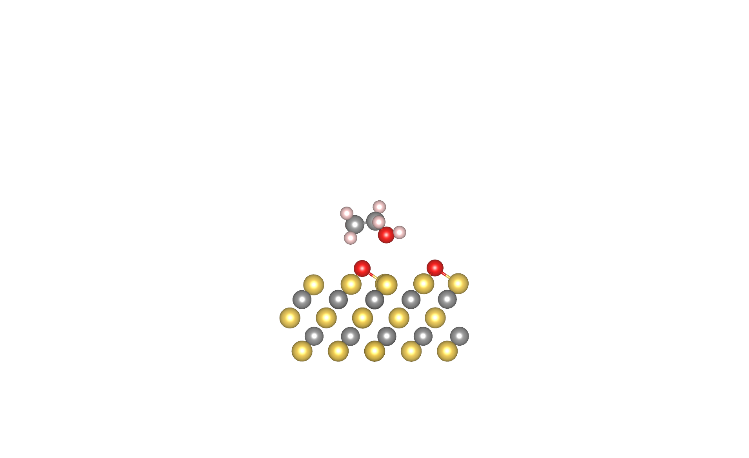  O-EtOH-O (-0.290 eV) | 2.6 |
|  | 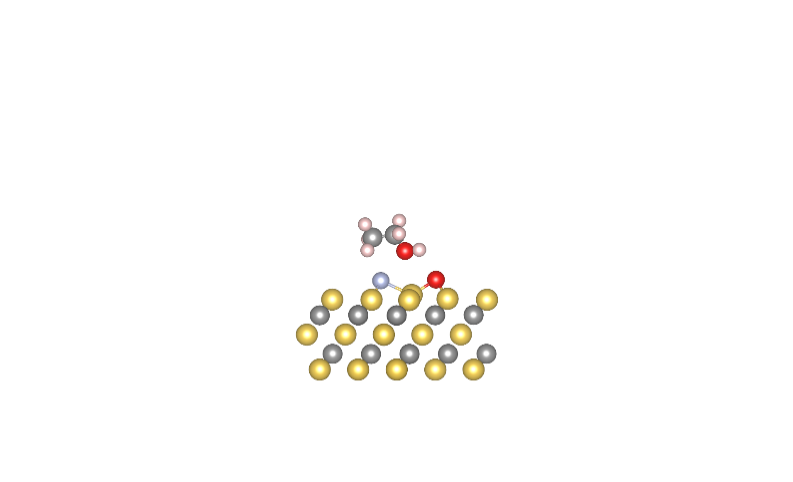  F-EtOH-O (-0.410 eV) | 3.7 |
| 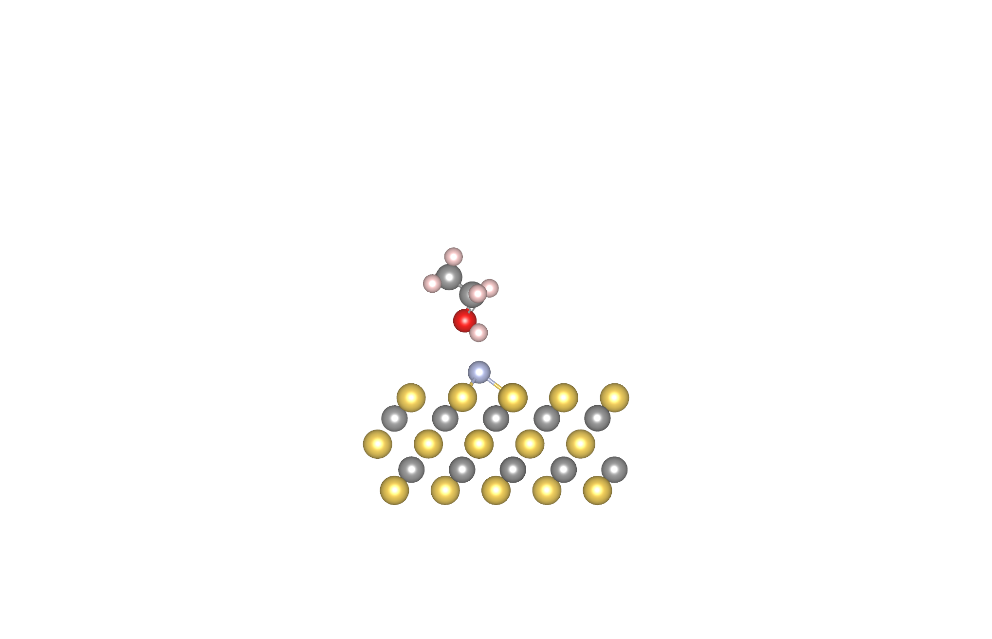  EtOH-F (-0.028 eV) | OH-EtOH-F (-0.990 eV)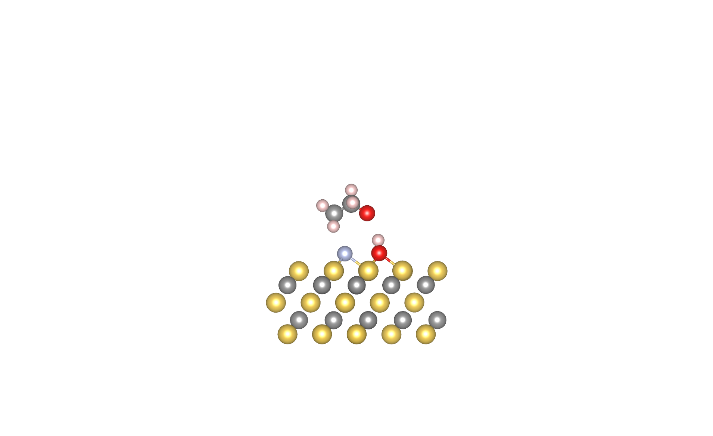 | 35.3 |
|  | O-EtOH-F (-0.410 eV)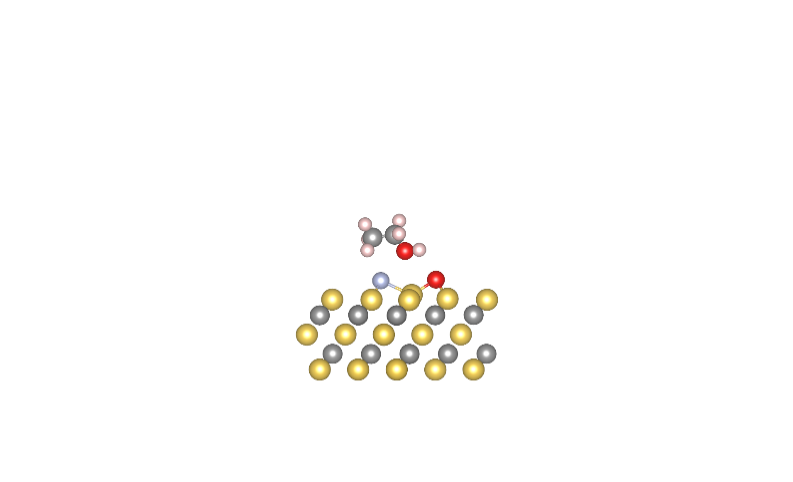 | 14.6 |
|  | F-EtOH-F (-0.230 eV)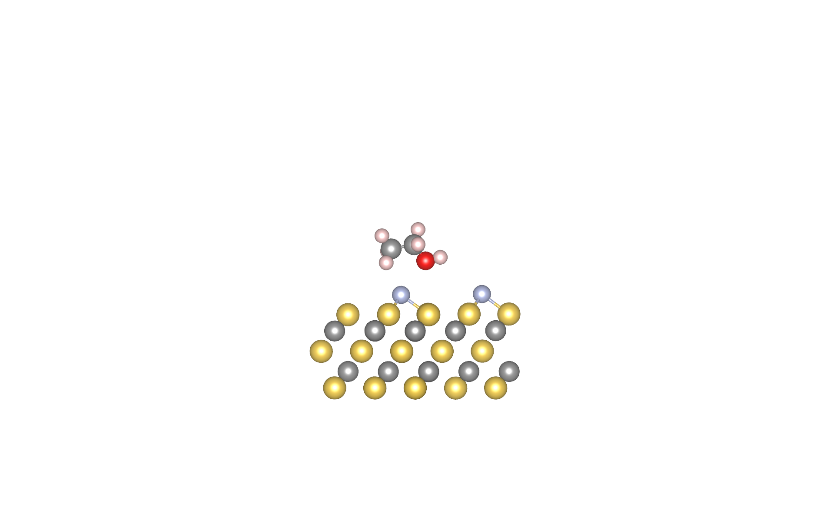 | 8.2 |

The comparison revealed the strengthening effect of one additional functional group on ethanol-MXene interaction. With one more group, either -OH, -O or -F, added into EtOH-OH pair, the rearranged pair would become chemically stable. With one more group added into EtOH-F pair, the binding energy of the rearranged pair could be enhanced up to 35 times.


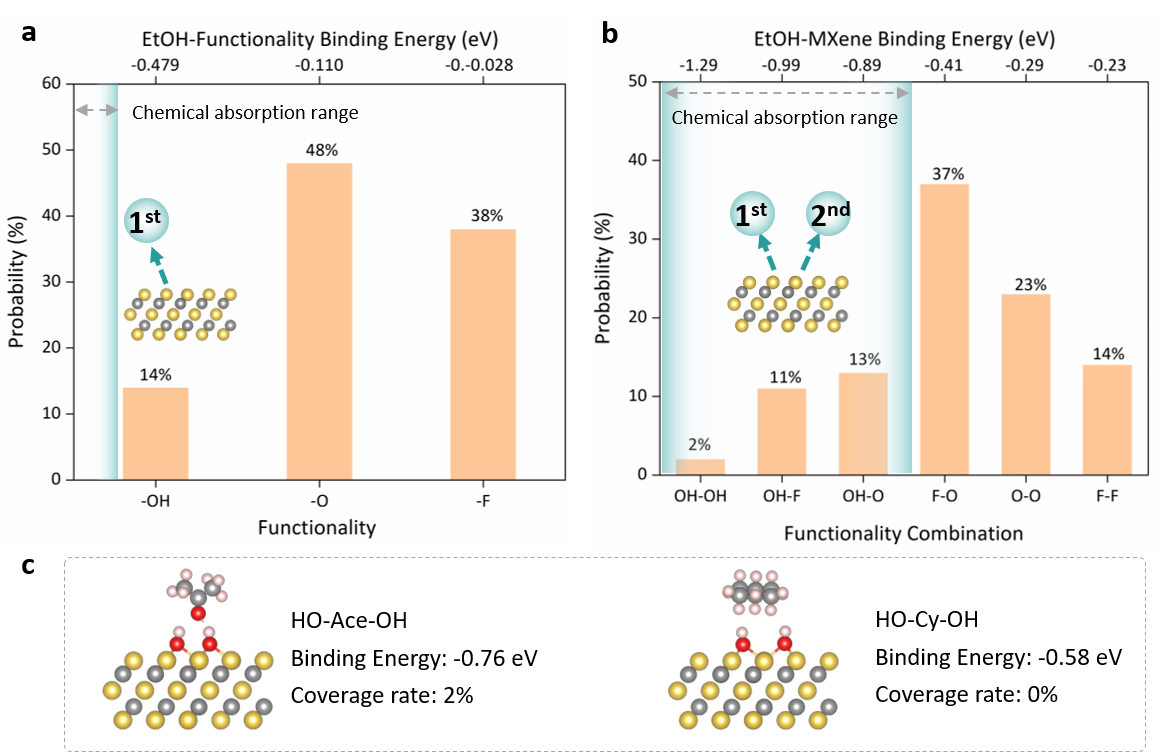
**Supplementary Fig. 11. Decoration coverage rate of Solvent-treated MXene membranes. Theoretical probability distribution of (a) functional groups and (b) functional pairs appearing on MXene surface. (c) Decoration coverage rate of Ace-M and Cy-M.**

Decoration coverage rate is here simplified as the percentage of Ti atoms with stable solvent decoration against all functionalized Ti atoms on MXene basal plane. Since this value can not be experimentally measured, it is alternatively obtained by carrying out an XPS- and density-functional theory (DFT)-based analysis in three steps, including (1) to figure out the functionality and/or functionality pairs that can contribute to a chemically stable solvent adsorption (E_abs_ > -0.62 eV); (2) to calculate their respective probability of existence on MXene surface; (3) to add up these values into an overall probability, which can theoretically translate into the decoration coverage. Moreover, by its molecular formula, Ti_3_C_2_O_0.66_(OH)_0.18_F_0.51_ used in this study determines the probability of each single group attached onto a random Ti atom (Supplementary Fig. 11a):

*P*(O) = 0.66 / (0.66+0.18+0.51) = 0.48

*P*(OH) = 0.18 / (0.66+0.18+0.51) = 0.14

*P*(F) = 0.51 / (0.66+0.18+0.51) = 0.38

Following these steps, we first examined the “One-to-One” decoration mode in EtOH-M. According to DFT results (Fig. 3d and Supplementary Table 2), neither -OH, -O or -F alone is able to form a stable EtOH-MXene combination.

We then studied the “One-between-two” decoration mode. Fig. 4a shows that when combined with OH-OH, OH-F, and OH-O, ethanol can stably decorate MXene surface. According to probability theory and MXene preparation mechanism, the functionalization of a random Ti atom and that of the neighboring Ti atom are two independent events. Therefore, *P*(O), *P*(OH) and *P*(F) should remain the same for both the 1^st^ and 2^nd^ atom, and the probability distribution can be calculated as below and recorded in (Supplementary Fig. 11b):

*P*(OH-OH) = *P*(OH_1_) × *P*(OH_2_) = 0.02

*P*(OH-F) = *P*(OH_1_) × *P*(F_2_) + *P*(OH_2_) × *P*(F_1_) = 0.11

*P*(OH-O) = *P*(OH_1_) × *P*(O_2_) + *P*(OH_2_) × *P*(O_1_) = 0.13

······

Therefore, the decoration coverage rate for EtOH-M is estimated to be 0.27 (27%). The same analysis is applied for Ace-M and Cy-M. DFT results demonstrate that the only pair leading to stable acetone-MXene combination is OH-OH while no such pair exists for Cy-M. Their decoration coverage rate is thus 2% and 0, respectively (Supplementary Fig. 11c). It is worth mentioning that the decoration coverage rate is not considered under “One-between-X” mode (X>2) as it is proved in Supplementary Fig. 10 that this mode cannot yield a stable solvent-MXene combination.


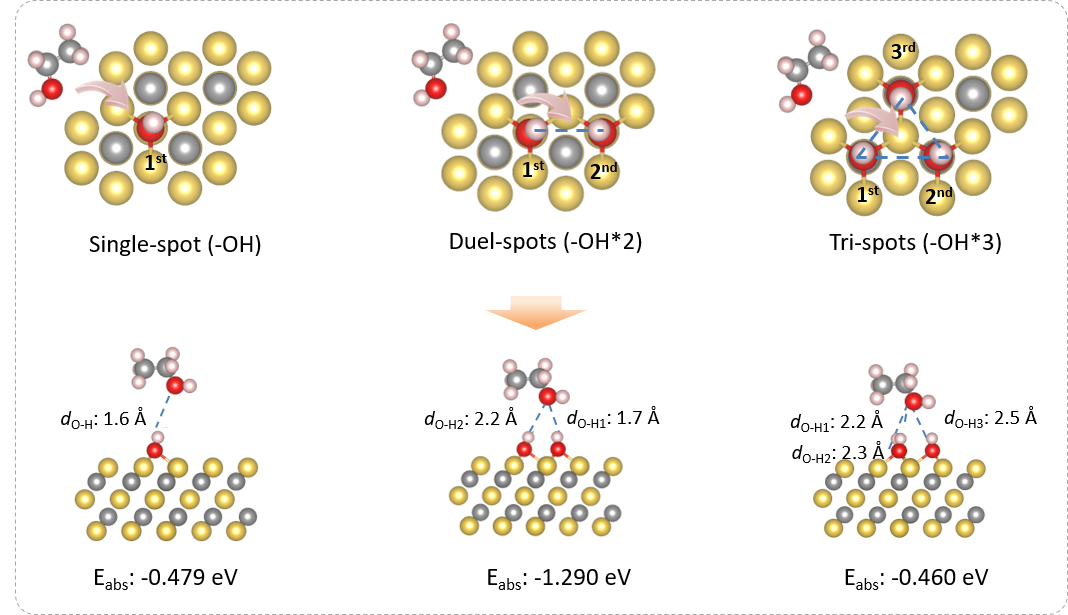


**Supplementary Fig. 12. Ethanol-MXene interactions in “one-to-one”, “one-between-two” and “one-between three” configurations (top) and corresponding optimal binding energy (bottom).**

When calculating the binding energy between ethanol and MXene via H-bond, we considered three possible configurations where one ethanol molecule was allowed to approach and stabilize near 1, 2 or 3 hydroxyl groups on MXene (M-OH). The interaction strength was found associated with the number of involving groups and ethanol-OH distance (*d*_O-H_, defined as the distance between O from ethanol and H from M-OH).

In 1-to-1 mode, *d*_O-H_ was 1.6 Å and E_abs_ was -0.479 eV. In 1-between-2 mode, ethanol stayed at a middle point between two -OH with *d*_O-H1_ and *d*_O-H2_ respectively being 1.7 Å and 2.2 Å, which yielded a much higher E_abs_ of -1.290 eV. However, with another -OH introduced, the most energetically favored position for ethanol was around the triangular center that was 2.2 Å, 2.3 Å and 2.5 Å away from the three -OH. The increased *d*_O-H1,_ *d*_O-H2_, and *d*_O-H3_ resulted in the decrease of overall E_abs_ to only -0.460 eV. This suggested the high dependence of ethanol-MXene affinity on their intermolecular distance, which is also a typical feature of H-bond. This also implied that the most stable positioning of solvent molecules on MXene surface would be between 2 groups rather than among 3 or more.


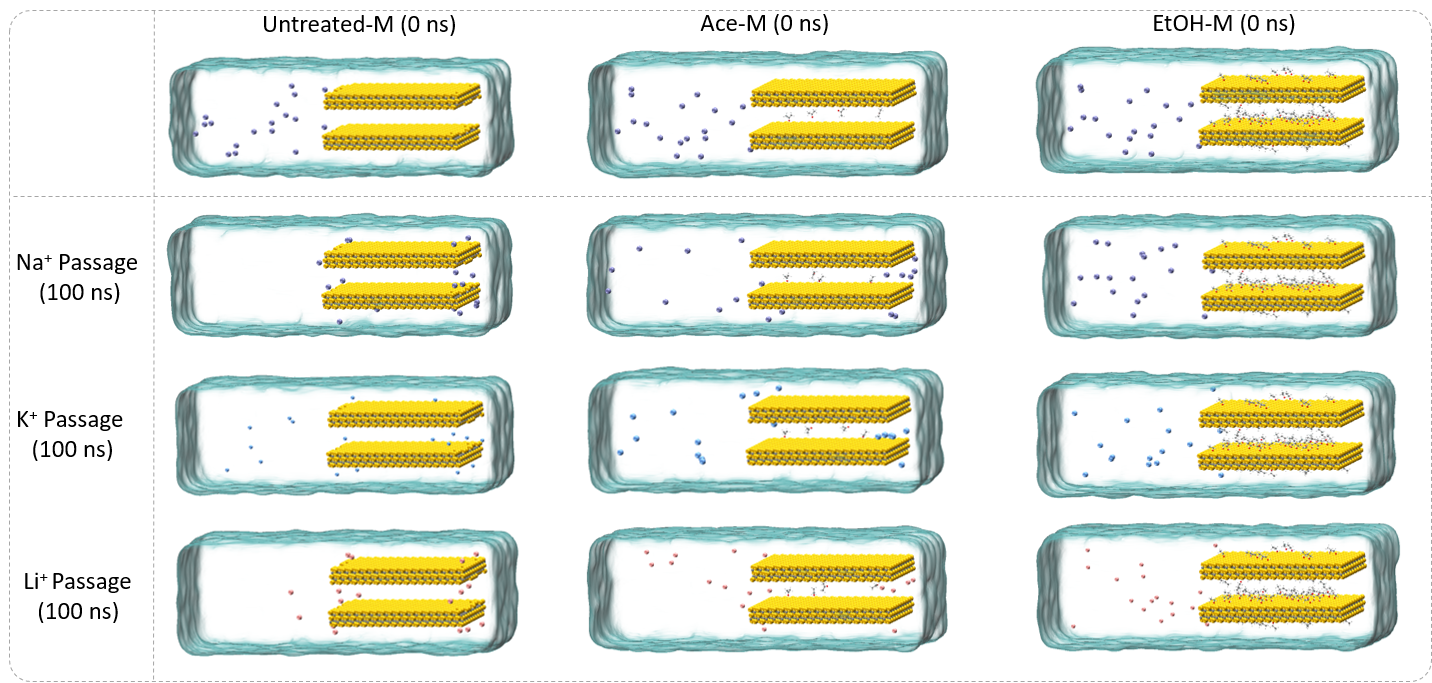


**Supplementary Fig. 13. Molecular dynamics (MD) simulations of Na^+^, K^+^ and Li^+^ passage through channels of Untreated-M, Ace-M and EtOH-M in 100 ns.**

The comparison of ions permeating different channels over a 100-ns time frame suggested that the decoration of solvent molecules could retard ionic transport. Such ion-sieving ability was found correlated to solvent coverage in MXene channels, which largely depended on the MXene-solvent binding probability and strength. While Untreated-M allowed free transport of Na^+^, K^+^ and Li^+^, Ace-M and EtOH-M would cause moderate and substantial transport rate decrease, respectively.


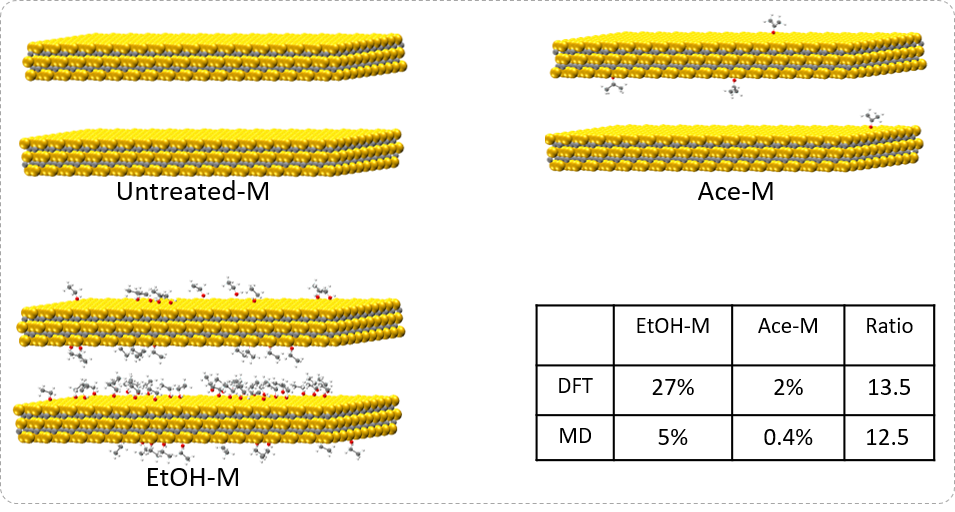


**Supplementary Fig. 14. MD simulations of MXene channels decorated by acetone and ethanol, and their decoration rate ratio compared to that shown in DFT simulation.**

The setup of solvent-treated MXene channels were completed in a similar fashion as in experiments, including solvent impregnation, stabilization and “evaporation” in the simulated channel. In particular, the “evaporation” step decided solvent decoration coverage rate of Ace-M and EtOH-M, and was achieved by applying a “solvent-MXene distance criterion” to eliminate unbound solvent molecules. Considering the short-range nature of H-bond nature and the bond length of C-OH and C=O, the criterion was set to be 2.3 Å and 2.3 Å for ethanol and acetone, respectively. This led to 5% coverage rate for EtOH-M and 0.4% for Ace-M. Although these values were lower than that summarized from DFT, the coverage rate ratio of EtOH-M against Ace-M from MD (12.5) and DFT (13.5) was highly consistent, largely proving the validation of both simulation methods.

We attributed the discrepant coverage rate to the limited MD simulated system that comprised only 20 (length) × 18 (width) × 2 (two sides) = 720 Ti atoms. To reach the theoretically maximal decoration rate as predicted in DFT, a much larger simulated area will be required.


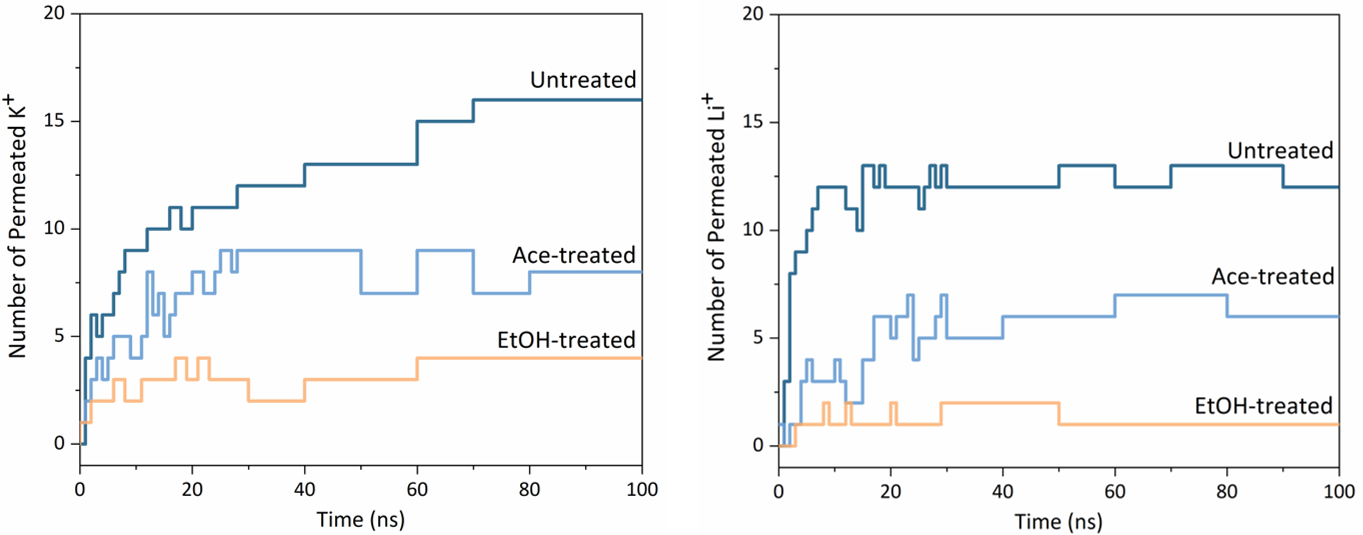


**Supplementary Fig. 15. MD simulations of K^+^ and Li^+^ passage through channels of Untreated-M, Ace-M and EtOH-M in 100 ns.**

Similar to the case of Na^+^, simulated K^+^ and Li^+^ showed varied permeation rate (*K*) in Untreated-M, Ace-M and EtOH and follow the order of *K*_Untreated-M_ > *K*_Ace-M_ > *K*_EtOH_. This suggested that the improved ion-rejecting ability of solvent-treated membranes work on different ions based on a size exclusion mechanism.

**Supplementary References**

1. Ding, L. *et al.* MXene molecular sieving membranes for highly efficient gas separation. *Nat. Commun.* **9**, 1–7 (2018).

2. Zhu, J. *et al.* Precisely tunable ion sieving with an Al13- Ti3C2Tx lamellar membrane by controlling interlayer spacing. *ACS Nano* **14**, 15306–15316 (2020).

3. Zhang, C. J. *et al.* Oxidation Stability of Colloidal Two-Dimensional Titanium Carbides (MXenes). *Chem. Mater.* **29**, 4848–4856 (2017).

4. Lu, Z. *et al.* Self-Crosslinked MXene (Ti3C2Tx) Membranes with Good Antiswelling Property for Monovalent Metal Ion Exclusion. *ACS Nano* **13**, 10535–10544 (2019).

5. Ding, L. *et al.* Effective ion sieving with Ti3C2Tx MXene membranes for production of drinking water from seawater. *Nat. Sustain.* **3**, 296–302 (2020).

6. Lu, Z., Wu, Y., Ding, L., Wei, Y. & Wang, H. A Lamellar MXene (Ti3C2Tx)/PSS Composite Membrane for Fast and Selective Lithium-Ion Separation. *Angew. Chemie - Int. Ed.* **60**, 22265–22269 (2021).

7. Qian, Y. *et al.* Enhanced Ion Sieving of Graphene Oxide Membranes via Surface Amine Functionalization. *J. Am. Chem. Soc.* **143**, 5080–5090 (2021).

8. Abraham, J. *et al.* Tunable sieving of ions using graphene oxide membranes. *Nat. Nanotechnol.* **12**, 546–550 (2017).

9. Jia, Z. & Shi, W. Tailoring permeation channels of graphene oxide membranes for precise ion separation. *Carbon N. Y.* **101**, 290–295 (2016).

10. Liu, N. *et al.* High-temperature stability in air of Ti3C2Tx MXene-based composite with extracted bentonite. *Nat. Commun.* **13**, 1–10 (2022).

11. Zhao, X. *et al.* Antioxidants Unlock Shelf-Stable Ti3C2Tx (MXene) Nanosheet Dispersions. *Matter* **1**, 513–526 (2019).

12. Habib, T. *et al.* Oxidation stability of Ti3C2Tx MXene nanosheets in solvents and composite films. *npj 2D Mater. Appl.* **3**, 1–6 (2019).
